# Neuro-symbolic NLP: taxonomy, assessment, and directions

**DOI:** 10.3389/frai.2026.1797587

**Published:** 2026-05-22

**Authors:** Stergios Chatzikyriakidis, Shalom Lappin

**Affiliations:** 1 Centre for Linguistic Theory and Studies in Probability, University of Gothenburg, Gothenburg, Sweden; 2 Department of Philology, University of Crete, Rethymno, Greece; 3 School of Electronic Engineering and Computer Science, Queen Mary, University of London, London, United Kingdom; 4 Department of Informatics, King's College London, London, United Kingdom

**Keywords:** compositionality, explainability, hybrid models, linguistic theory, neuro-symbolic, NLP, reasoning, transparency

## Abstract

Neuro-symbolic (NeSy) approaches promise to overcome the limitations of purely neural and purely symbolic NLP. In this paper we survey recent development in NeSy NLP and propose a system classification framework that combines Kautz's integration types with Lappin's injective-federative distinction. We then apply this taxonomy and show that even though federative architectures consistently outperform injective approaches, they remain underexplored. We assess performance across compositional generalization, reasoning, and robustness benchmarks, examine existing attempts at linguistic theory integration, and show how the taxonomy can guide future architectures that preserve the strengths of formal linguistic frameworks. We conclude with directions for scaling federative systems and exploring tighter integration.

## 1 Introduction

It is undeniable that Neural models of language have achieved remarkable progress in the last decade or so. However, they still often rely on superficial patterns and also struggle with compositional generalization and systematic reasoning. On the other hand, symbolic approaches while offering transparency and strong generalization, lack scalability. *Neuro-symbolic* (NeSy) NLP promised to combine these strengths.

Different taxonomies have been proposed in an effort to organize the NeSy research landscape. Some focus on functional capabilities, classifying systems according to the research problems they address: knowledge representation, learning and inference, or explainability (Colelough and Regli, 2025). Others focus on architectural organization. Kautz (2022) proposes a six-type classification based on the depth of integration between neural and symbolic components. Lappin (2025) offers a simpler but architecturally grounded distinction between injective systems, which embed symbolic content within neural architectures, and federative systems, which maintain autonomous modules with structured interfaces. In this paper, we propose a unified classification framework that combines the existing taxonomies and survey the current landscape of NeSy NLP systems using this new combined taxonomy. We then examine existing attempts at linguistic theory integration, show how the taxonomy can guide linguistically-informed architectures, and propose directions for developing foundational neuro-symbolic NLP.

### 1.1 Kautz's taxonomy

**Figure 1:**
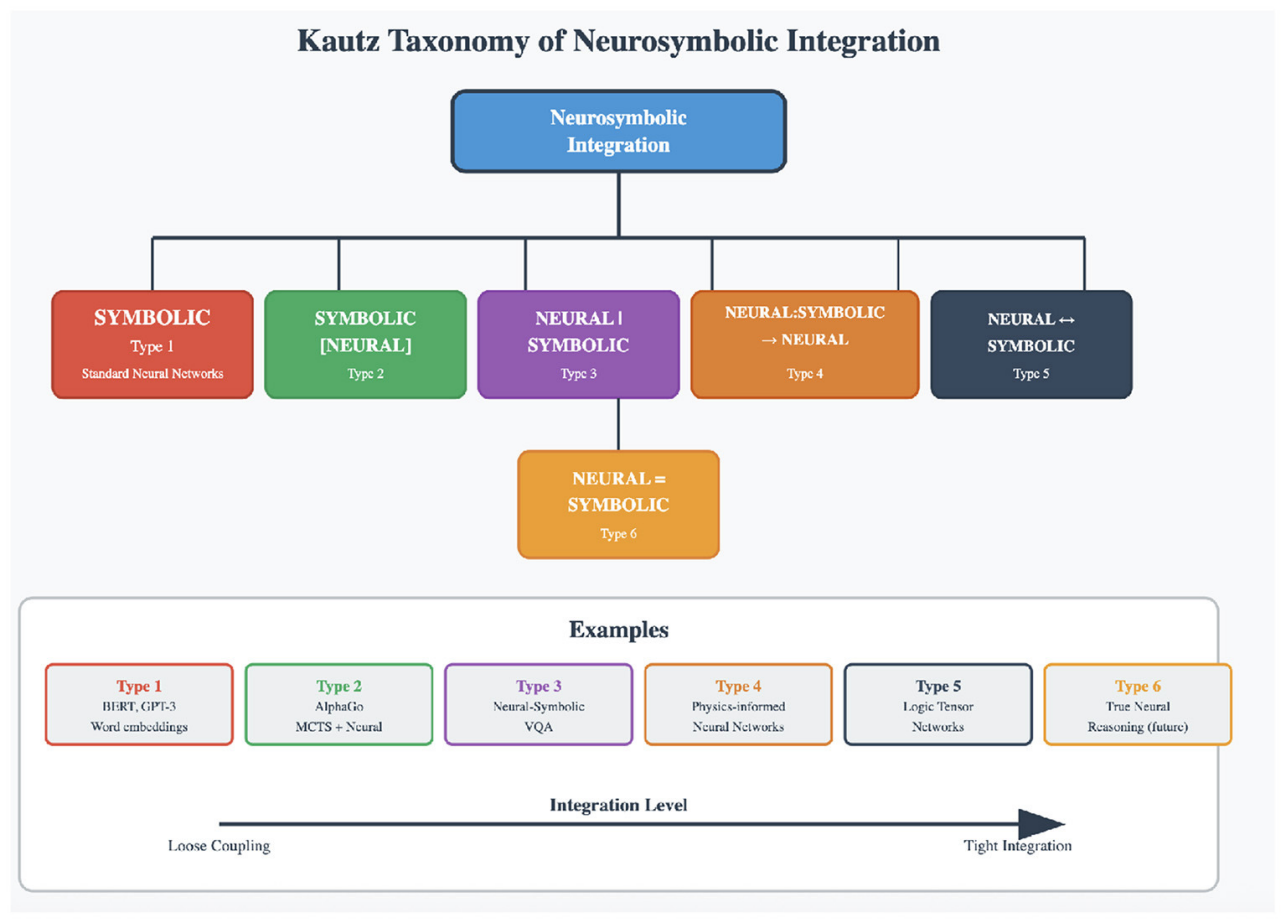
Kautz's taxonomy of neuro-symbolic architectures, showing the progression from loosely coupled systems (Types 1–2) to tightly integrated architectures (Types 5–6).

Kautz (2022) proposes a six-way classification of NeSy systems based on the level of integration between the neural and the symbolic components. Type 1 systems are standard neural networks, in which the symbolic input is turned into vector representations for neural processing and then mapped back to symbolic output. Type 2 systems embed neural components within symbolic problem solvers. In these systems symbolic techniques invoke neural methods. AlphaGo is an example, where Monte Carlo tree search (symbolic) calls neural networks to evaluate game positions. In Type 3 systems, neural networks interpret perceptual data as symbols that are then processed by symbolic reasoners. Examples include the Neuro-Symbolic Concept Learner (Mao et al., 2019) and NS-VQA (Yi et al., 2018). Type 4 systems involve the use of symbolic reasoning to generate or label training data for neural networks, as in physics-informed neural networks. The last two types, Types 5 and 6, involve the most tightly connected systems. Type 5 systems like Logic Tensor Networks (Serafini and Garcez, 2016) and Neural Theorem Provers (Rocktäschel and Riedel, 2017; Shah and Zadrozny, 2026) attempt bidirectional interaction. There, symbolic operations are represented as tensor operations that can be optimized via gradient descent. Type 6 represents true unification of neural and symbolic processing and remains largely aspirational. However, on a conceptual level, despite the move toward tighter integration, even the most advanced existing systems (Type 5) still maintain a conceptual distinction between the neural and the symbolic components. They treat them as distinct systems that must be connected in some way (Figure 1).

### 1.2 Lappin's injective vs. federative distinction

Lappin (2025) makes a proposal that is based on a simpler distinction, is architecturally grounded, and cuts across Kautz's types. On the one hand, we have injective architectures that insert symbolic representations directly into the processing operations of a Deep Neural Network (DNN). This injection can happen in a number of ways: (1) via modifications in the architecture that incorporate symbolic bias in the computation, (2) using knowledge enrichment in which a symbolic system generates a biased training distribution (Hinton et al., 2015), and (3) via symbolic markers injected in the training. DNNs that incorporate syntactic structure into LSTMs (Tai et al., 2015; Bowman et al., 2016; Yogatama et al., 2017) or transformers (Bai et al., 2021; Sachan et al., 2021), and CNNs enriched with handcrafted knowledge features for domain-specific tasks (Zeng et al., 2014; Nguyen and Grishman, 2015) are some examples. On the other hand, a *federative* model combines a neural architecture with a symbolic module as part of the system, in which each module retains its independence. The neural component extracts features for an interface that labels them and then sends them to the logic component, thus, keeping the computational integrity of both systems (Cunnington et al., 2023).

Lappin shows an asymmetry w.r.t the performance of these two approaches in the taxonomy. On the one hand, injective models produce small performance gains over their non-enriched counterparts. To give an example, tree-enriched versions of BERT and RoBERTa show 1%–2% accuracy increases on the GLUE benchmark (Bai et al., 2021), and enrichment with dependency graphs shows gains of 2.6–3.5 F1 scores for semantic role labeling, though these improvements require gold parses and largely disappear with predicted parses (Sachan et al., 2021). These already marginal gains seem to diminish further when DNNs receive more training data. Furthermore, the transparency claims often made for injective models also remain questionable, given that these systems still use non-linear activation functions (e.g., ReLU and softmax). On the other hand, Lappin notes, federative models show more substantial advantages in performance. For example, Cunnington et al.'s (2023) Feed Forward Neural-Symbolic Learner (FFNSL) presents considerable performance boost over non-symbolic baselines on difficult image classification tasks, requires substantially less training data to reach high accuracy, exhibits greater robustness under distributional shift, and generates transparent, inspectable rule-based hypotheses.^1^ Lappin further hypothesizes that this asymmetry between the two approaches (injective vs. federative) may actually come from fundamental differences in how DNNs and symbolic systems represent patterns of regularity. It could be the case that DNNs may be unable to easily integrate symbolic components into their distributed representations (Smolensky, 1990).

### 1.3 Comparative analysis

The two taxonomies, Kautz's on the one hand, and Lappin's on the other, focus on complementary aspects of NeSy systems. Kautz's taxonomy is mostly concerned with the mechanism of interaction between the two components, for example the way information flows between components, while Lappin puts the architectural location of symbolic content, i.e., whether it lives inside or outside the neural network, at the core of the taxonomy. As already mentioned, there are other ways of organizing NeSy systems. Colelough and Regli (2025) propose a taxonomy that classifies NeSy systems based on the research problems they are called to tackle: knowledge representation, learning and inference, logic and reasoning, explainability and trustworthiness, and meta-cognition. Such classifications prove valuable to identify research gaps in what NeSy systems can do, but they provide limited guidance on how to design systems to achieve those capabilities. We believe that architectural taxonomies like those of Kautz and Lappin offer more direct design insights by revealing which integration methods produce gains, and which face architectural limitations. The synthesis we propose aims to put together these architectural perspectives and, at the same time, remain grounded in specific challenges.

**Figure 2:**
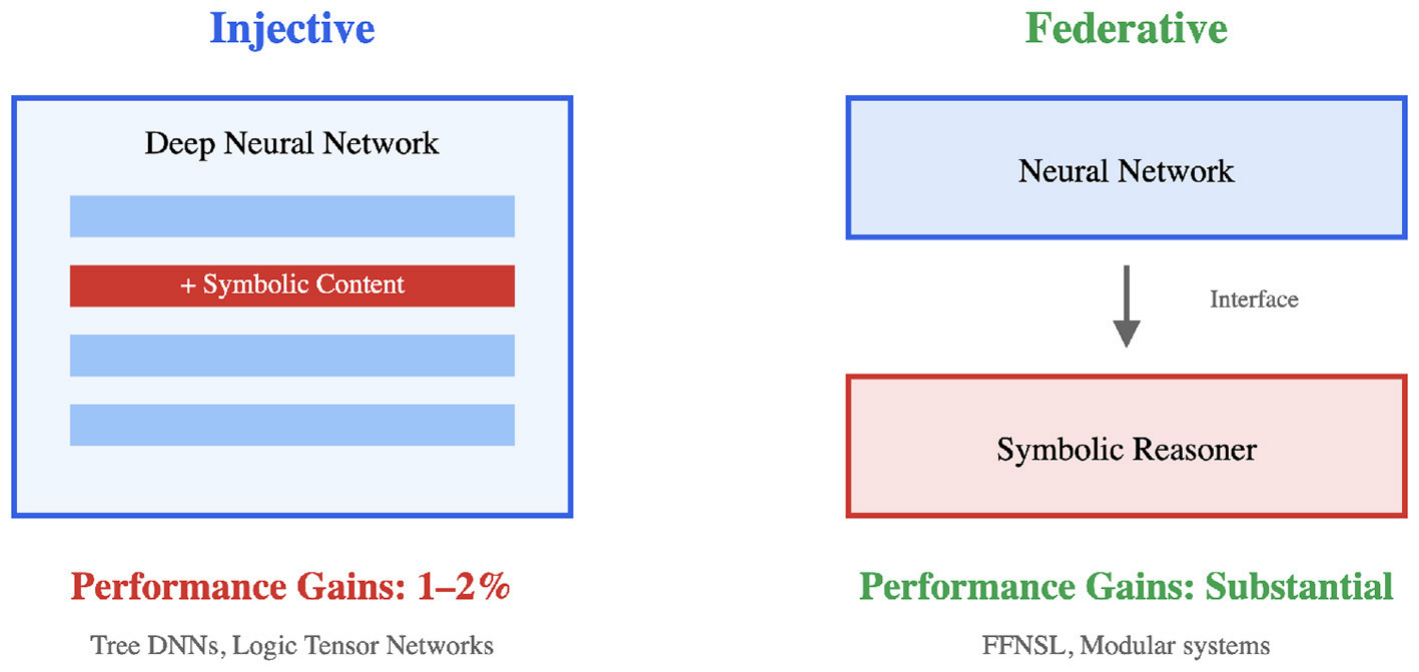
An overview of Lappin's taxonomy: on the left we see Injective systems (**left**) showing marginal gains (1%–2%) and tight integration, while on the **right** we see federative systems showing substantial improvements via modular autonomy. The asymmetry might point to constraints on how the neural component absorbs symbolic knowledge.

Table 1 presents the Chatzikyriakidis-Lappin (CL) taxonomy. It is a two-dimensional classification system combining Lappin's taxonomy with five interaction modes we have identified in the literature. Systems are organized by architectural location first (Injective vs. Federative), interaction mode second. In this way Kautz's types may appear non-consecutively. For example, Type 4 systems, i.e., systems in which symbolic reasoning generates or labels training data for neural learning, are split into I-T (symbols embedded then discarded), I-G (symbolic data generation for self-training), F-T (external symbolic preprocessing), and F-G (external symbolic data generation). Similarly, Type 3 systems, in which neural networks produce symbolic representations for symbolic reasoning, are split to I-R (internal conversion) and F-R (external reasoner). This non-sequential ordering is a choice, given that we privilege architectural location over the interaction mechanism. This is because we believe that Lappin's taxonomy reveals more about system performance than Kautz's integration depth. The first dimension indicates architectural location (Injective or Federative), while the second interaction mode: Teaching (symbolic knowledge transferred then discarded), Constraining (symbolic rules shape neural computation), Generating (symbolic systems produce training data), Reasoning (neural outputs feed symbolic inference), and Loop (dynamic bidirectional interaction) (Figure 2).

**Table 1:** The CL system.

CL code	Related Kautz type(s)	Description	Example system
Injective Hybrids (I-*): symbols embedded within neural architecture			
I-T	Type 4	Symbolic knowledge distilled into neural weights, then discarded	Pre-training LMs with syntactic objectives
I-C	Type 5	Symbolic rules compiled into network architecture	Logic Tensor Networks
I-G	Type 4	Symbolic system generates data for neural self-training	Grammar-based data augmentation
I-R	Type 3	Internal neural representations converted to symbols for reasoning	Neural semantic parser + theorem prover
15.6-2.4,-1499ptI-L	Type 5	Bidirectional symbolic-neural interaction within the model	Logical Neural Networks
Federative Hybrids (F-*): external symbolic modules interfacing with neural components			
F-T	Type 4	External symbolic preprocessing creates neural training data	Rule-based feature extraction + neural classifier
F-C	Type 2	External symbolic constraints guide neural processing	Constrained neural text generation
F-G	Type 4	External symbolic system generates training data	Knowledge graph-based dataset creation
F-R	Type 3	Neural frontend feeds external symbolic reasoner	NLProlog, DSR-LM
F-L	Type 2/3	Dynamic interaction between separate neural and symbolic modules	PAL, SatLM

It combines Lappin's architectural dimensions with Kautz's interaction patterns. Note that the Kautz column indicates approximate correspondences; see text for discussion of limitations.

I, Injective (symbols within neural architecture); F, Federative (external symbolic modules); T, Teaching; C, Constraining; G, Generating; R, Reasoning; L, Loop.

The majority of existing NeSy work falls into the injective categories (I-*), i.e., to those employing various mechanisms of embedding the symbolic content within neural architectures. However, these are the approaches that, in the examples Lappin considers, show only marginal improvements, typically within the 1%–2% accuracy range and further diminish with additional training data. Federative approaches, which, in the cases that he cites, indicate substantially stronger performance, are less explored, especially at foundational model scale. This state of affairs might point to the fact that the field has invested in architectural integration strategies that may face fundamental limitations, while leaving more promising paradigms underdeveloped. In Section 2, we use this taxonomy to systematically review major NeSy NLP systems of the last decade, documenting how architectural choices shape empirical performance.

### 1.4 How to read the Chatzikyriakidis-Lappin (CL) code

A tag such as F-L has two characters:

- The first (I or F) indicates *where the symbols live*, i.e., inside or outside the model (Injective/Federative).
- The second (T, C, G, R, or L) describes the *interaction mode*, a categorization we propose based on patterns observed across the NeSy literature, loosely related to but not identical with Kautz's types.

For example, I-T refers to an injective model, in which symbolic training data are taught once then discarded, whereas F-L refers to a federative model involving a live bidirectional loop at inference time.

Of course, our taxonomy is not without limitations. We identify mainly two. First, the correspondence of interaction modes and the types proposed in Kautz is not exact. This is because Kautz's taxonomy measures integration depth, while we aim to capture information flow patterns. Furthermore, the mapping of the two dimensions in the new taxonomy can also be difficult to make clear in some cases, particularly when models exhibit hybrid characteristics. Furthermore, we cannot adequately distinguish the depth of integration. For example, an injective system that only adds syntactic features to input embeddings is not the same as a system that rebuilds its attention mechanisms based on type-theoretic constraints. However, the two systems receive the same classification in this taxonomy. Last, and perhaps most important, the taxonomy assumes a clear boundary between the symbolic and the neural component. However, systems implementing differentiable proof systems built directly into transformer blocks, or systems keeping hybrid representation spaces encoding both distributional and logical semantics resist a clear border between neural and symbolic components.

## 2 Landscape of neuro-symbolic NLP systems

In this section, we review a number of major NeSy NLP systems. We organize the discussion according to the CL classification framework introduced in Section 1.3 and examine the way architectural choices (injective vs. federative) and interaction mechanisms shape system capabilities.

### 2.1 Injective systems: symbol embedding in neural architectures

#### 2.1.1 Injective-Constraining (I-C): compiled symbolic structure

Injective systems embed symbolic constraints directly into the neural system. This is done via structural modification or differentiable logical operators.

Logic tensor networks (LTN) (Badreddine et al., 2022) encode end-to-end differentiable first-order logic via the integration of fuzzy logic directly into the computational graph, with predicates being represented as neural networks, constants as learnable embeddings, and logical connectives as differentiable fuzzy operators. LTN uses Real Logic, a differentiable form of first-order logic, to perform tasks such as multi-label classification and relational learning.

Riegel et al. (2020) propose Logical neural networks (LNN), a system, where neurons are directly mapped to weighted real-valued logic, allowing, at least theoretically, for bidirectional inference. However, when applied to question answering (Kapanipathi et al., 2021), the theoretical promise does not hold up completely. While the full NSQA pipeline achieves state-of-the-art on QALD-9 and LC-QuAD 1.0, the LNN reasoner's isolated contribution is modest (approximately 3 F1 points over a deterministic SPARQL baseline on LC-QuAD). This is in line with Lappin's critique of injective approaches that argues that even if you compile symbolic constraints into the network, you are essentially guiding the learning process, and not fundamentally changing the distributed representations.

DeepProbLog (Manhaeve et al., 2021) integrates neural predicates into ProbLog's probabilistic logic programming framework. Neural networks act as predicates for perceptual tasks. DeepProbLog compiles the logic program into arithmetic circuits enabling the system to compute gradients efficiently. This achieves strong sample efficiency: the system needs just hundreds of training examples rather than the tens of thousands needed by pure neural baselines.

The performance increases show I-C's real potential: when logical structure is infused into the network, compositional generalization can emerge with only minimal examples, given that the composition rules are learned and not memorized. Additionally, for tasks like visual question answering, these systems are able to systematically generalize to novel combinations of learned concepts. However, the issue of scaling is still challenging. Given that the probabilistic inference overhead grows as program complexity grows, these systems are limited to small-scale problems.

Scallop (Li et al., 2023) presents an I-C integration infrastructure using a Datalog-based NeSy programming language that further enables differentiable reasoning. Tree-LSTMs (Tai et al., 2015) use syntactic structure via the incorporation of parse trees into the LSTM. The system performs well on benchmarks where syntax is a key predictor, such as the Stanford Sentiment Treebank 5-class (SST-5) benchmark (51.0 vs. 46.4%). However, it falls behind on tasks where syntactic structure offers less leverage. Syntax-aware transformers (Bai et al., 2021; Sachan et al., 2021) show a similar picture with a 0.3%–2.2% increase on various benchmarks, thus following the general pattern. Neural Logic Machines (Dong et al., 2019) learn first-order logic rules via neural tensor operations. They manage to get perfect generalization on synthetic reasoning tasks (family trees, sorting, and shortest paths). Despite this, the system remains limited to domains with explicit logical structure.

Generalizing, one could argue that for I-C systems, symbolic structures provide decisive advantages w.r.t. compositional generalization, in which explicit structure directly encodes the target abstraction. We have seen cases where this is true for example, DeepProbLog's order-of-magnitude sample efficiency and CPG's 1,000 × on SCAN. At the same time, this type of systems provide only small improvements (1%–2%) on more general NLU tasks, where symbolic constraints compete with the network's statistical shortcuts.

#### 2.1.2 Injective-Teaching (I-T): distilled, then discarded symbolic knowledge

Systems of this type insert symbolic knowledge in neural weights during pre-training, and then lose the symbolic component. This means that the advantages symbolic structure has to offer early in training disappear as the optimization continues.

Syntax-aware pre-training objectives, such as those applied to variants of BERT and RoBERTa, are examples of such architectures. The accuracy gains are modest with Tree-enriched BERT variants showing between 1 and 2% accuracy increase on GLUE (Bai et al., 2021). Furthermore, given the nature of the architecture, these already small increases often disappear when additional training data are given to the models. This suggests that a symbolic backbone can help early in the training process, as it provides useful inductive biases, but, nonetheless, it fails to provide a persistent architectural advantage. The reason is that gradient descent will eventually discover equivalent representations via purely distributional patterns, making the initial symbolic injection irrelevant.

I-T systems are thus distinct from other injective approaches architecturally, given that they abandon the symbolic component entirely after training has been done. This is in contrast for example to I-C systems that maintain compiled symbolic constraints throughout deployment, or I-R systems that generate symbolic representations at inference time.

#### 2.1.3 Injective-Reasoning (I-R): internal NeSy conversion

In this category, we find architectures in which neural networks produce intermediate symbolic representations, that are processed via reasoning mechanisms in a unified, end-to-end fashion. The PRover (Saha et al., 2020) is a good example of such architecture. The transformer in this system predicts both the answers and the proof graphs. Neural components generate the nodes and edges that represent facts, rules, and logical dependencies. These attributes allow Scallop (Li et al., 2023) to utilize a Datalog-based language for differentiable reasoning within I-C integration. Tree-LSTMs (Tai et al., 2015) take a different approach by embedding syntactic parse trees into the model state. This design improves performance on syntax-heavy tasks like SST-5 (51.0 vs. 46.4%), but the benefit is lost when grammar is not a primary predictor. Syntax-aware transformers (Bai et al., 2021; Sachan et al., 2021) mirror this pattern, yielding only marginal gains between 0.3 and 2.2%. Neural Logic Machines (Dong et al., 2019) use tensor operations to learn first-order logic, reaching perfect scores on synthetic tasks, like sorting or path finding while remaining restricted to rigid logical domains. Although differentiability facilitates the joint optimization of perception and reasoning, these systems still lack true symbolic interpretability. Their representations are learned latently, and they prioritize correlation with training labels over any strict adherence to logical truth.

Other I-R systems are similar in this manner. For example, ProofWriter (Tafjord et al., 2021) uses T5 to iteratively produce single-step implications, gathering symbolic proof chains in one unified architecture. RNNLogic (Qu et al., 2021) makes use of logic rules as latent variables. An RNN-based generation system produces candidate rules and a reasoning module evaluates them. Rules are generated internally, the model uses them to reach an answer, but these rules are not shown to the user.

These systems allow for the joint optimization of perception and reasoning, which preserves differentiability, but they lack real symbolic interpretability. The representations are only learned latently. This means the model optimizes for correlation with training labels, rather than following any actual logical truth. This puts I-R systems in an awkward position, since they suffer from the high computational cost of maintaining symbolic structures, but lack the transparency of external reasoners. It is unsurprising, then, that F-R systems that involve a cleaner architectural separation, tend to supersede them.

#### 2.1.4 Injective-Loop (I-L): bidirectional internal interaction

The I-L is arguably the most ambitious injective approach, as it strives for bidirectional information flow between the neural and the symbolic components in a unified, trainable architecture. Think of it as trying to get the best of both worlds by having symbols and neurons talk to each other continuously during both training and inference.

NeurASP (Yang et al., 2020) takes a different route and embeds neural networks into Answer Set Programming. Neural perception is informing symbolic rules, and a number of symbolic constraints guide neural learning, all in a unified optimization loop. Differentiable Neural Computer (Graves et al., 2016) maintains both distributed and symbolic-like memory representations with bidirectional read-write operations.

The computational challenges involved in maintaining a tight bidirectional coupling between the two components have proven difficult to overcome as the scale of the data and the task increase. As such, it comes as no surprise that large-scale NLP implementations in this category remain rare. These systems work nicely on small, structured problems, but moving to foundation-model size is very difficult to achieve, as overhead grows faster than the actual benefits, and the optimization becomes unstable.

### 2.2 Federative systems

#### 2.2.1 Federative-Reasoning (F-R)

Probably the most successful category of federative systems pair the neural component with an external symbolic reasoner. A clear separation of the two components exist and neither component compromises its computational identity.

An example of such a system is NLProlog (Weber et al., 2019). In this system, sentences from a knowledge base are embedded via Sent2Vec. Then, when the system is asked to provide an answer, a Prolog theorem prover with weak unification searches for logical derivations. However, instead of the exactness of symbolic pattern matching, NLProlog uses nearest-neighbor lookup to discover relevant facts. In this way, the system is able to deal with the noise and variation of language, and yet remain logically sound.

On MedHop, a dataset that tests multi-hop reasoning abilities, NLProlog achieves 29.3% accuracy on hidden test, as well as fully interpretable proof traces. This means that one can see exactly the facts the system retrieved and the way these were chained together to reach a conclusion, something which is not possible in pure neural approaches.

DSR-LM (Zhang et al., 2023) builds on this architecture and provides even better results. The system uses a language model for perception, with weighted rule-based reasoning. The neural component deals with natural language, extracting symbolic predicates, and then the symbolic component applies a number of learned weighted rules in order to deal with deduction. The system surpasses pure neural baselines by more than 20% on tasks that require deductive reasoning. The clear separation of the two modules lets each one do what it does best, i.e., the LM deals with the noise and variation of linguistic input and the symbolic module handles inference.

Other F-R systems. Logic-LM (Pan et al., 2023) maps natural language to FOL or SAT constraints for solver refinement, boosting performance by 39.2% over standard prompting. LINC (Olausson et al., 2023) shows a comparable pattern. In that work, a StarCoder-15B model, integrated with a first-order logic prover beats GPT-4 on ProofWriter by 10%. Greedy NTP (Minervini et al., 2020) addresses link prediction using a hybrid of k-NN search and Prolog-style backward chaining. This setup achieves competitive WN18 accuracy while operating at significantly higher speeds than typical symbolic baselines. Beyond these applications, recent work has begun to target the challenges in these domains. It applies these neuro-symbolic techniques to the challenges of complex geometry and automated theorem proving. For example, Alpha Geometry (Trinh et al., 2024) combines a neural model for suggesting constructions with a symbolic deduction engine. In LEGO-Prover, (Wang H. et al., 2024) follow a different route. They use libraries of verified lemmas, resulting in the improvement of neural theorem proving on the miniF2F validation set by 9.0% (4.5% on test).

The pattern across F-R systems is clear and consistent. For tasks requiring a separation of perception from logical inference, federative systems present clear advantages (20%+ for DSR-LM, interpretable proofs for NLProlog). The architectural boundary emerges as a strength, given that each component can be optimized independently, verified separately, and also understood in isolation.

#### 2.2.2 Federative-Loop (F-L): dynamic iterative refinement

F-L architectures include federative systems that involve dynamic interaction between separate neural and symbolic modules during inference. These systems are also bidirectional in that the neural output informs symbolic reasoning, and symbolic results guide the neural generation.

Symbolic Working Memory (Wang S. et al., 2024) is an example of such an approach that is used for multi-step deductive reasoning. The system includes an external memory module that stores facts and rules in dual format (natural language and symbolic). When reasoning about a problem, the neural component parses the question and queries the memory for relevant information. Retrieved facts are converted to symbolic form, the symbolic reasoner performs rule grounding, and the results feed back to the neural component, which may query the memory again or generate intermediate conclusions.

This process continues until an answer is produced. In each step the symbolic representations in memory get updated, allowing the construction of chains of reasoning in an incremental fashion, instead of trying to deal with everything in one pass. The fact that the system involves a clear separation between neural understanding (which facts are relevant) and symbolic reasoning (how to connect these facts) has the welcome benefit that the components can be inspected and corrected independently.

F-L represents the most sophisticated federative architecture, as it involves a dynamic interaction between separate neural and symbolic modules at inference. This is not just one-way information flow, but a loop in which the neural output informs the symbolic component. Similarly, symbolic results guide neural generation, with the process iterating toward convergence.

Other F-L systems. Iterative refinement is also central to work like Natural Language Embedded Programs (NLEP) (Zhang et al., 2024), which targets formal reasoning. It generates and then executes Python programs step-by-step, improving interpretability. This code-driven approach echoes Program-Aided Language models (PAL) (Gao et al., 2023), where offloading reasoning into code improved over PaLM-540B on GSM8K by 15%. Satisfiability-Aided Language Models (SatLM) (Ye et al., 2023) takes this further and applies it to constraint satisfaction problems, using declarative constraints for SAT/SMT solvers, improving upon program-aided LMs by 23% on a subset of the GSM arithmetic reasoning dataset. Most recently, Symbolic Verification systems (e.g., Logic-Scaffold) (Quan et al., 2024) have closed the loop completely. They use external verifiers that check coherence in LLM-generated specs, with the resulting feedback fed back to shape subsequent corrections. F-L architectures have also proven effective for phonologically-grounded rhyme generation. Chatzikyriakidis and Natsina (2026) present a NeSy system for poetry written in Modern Greek that combines LLMs and a deterministic phonological engine in a Generate-Verify-Refine loop. Pure LLM generation achieves under 4% validity for phonologically correct poems, whereas the verification loop raises this to 73.1%. This makes clear that iterative symbolic feedback is effective, in cases where phonological constraints are needed. F-L systems shine when tasks call for multi-step verified reasoning. This is to be expected given their self-correcting ability. The capacity to reason across iterations lets them tighten their logic and clean up symbolic representations, which single-pass I-R and F-R models are unable to do. Naturally, there is a cost. Running multiple neural-symbolic round trips proves computationally expensive. But, when moving to critical domains, F-L architectures offer the necessary balance of capability and verification.

#### 2.2.3 Federative-Generating (F-G): external symbolic system generating training data

In F-G architectures, one finds symbolic modules as training data generators for neural components, i.e., they involve a symbolic generator that makes sure the generated data satisfies formal requirements. The neural component then learns from this verified dataset.

Neuro-Symbolic Data Generation (NSDG) (Li et al., 2024) is a system in this category that converts math problems to Satisfiability Modulo Theories Library (SMT-LIB) format (the standard format used for writing logic formulas for SMT solvers), and it mutates them systematically. These mutations preserve logical correctness, and at the same time are able to create diverse training instances. Examples in the resulting datasets have formal guarantees of logical validity. This is very useful for domains in which training data might be scarce, but formal specifications are available.

F-G and I-T systems are foundationally different in the way symbolic knowledge post-distillation is handled. I-T models discard the symbolic component after pre-training, but F-G models keep the generator active during the whole process. This allows for the on-demand synthesis of new training examples, a vital feature for adapting to shifting problem distributions. Current dataset expansion methods rely on neural techniques like back-translation or generative paraphrasing, which often produce plausible but strictly unverified results. F-G offers an alternative by ensuring that all augmented data meets formal, domain-specific requirements. Choosing these systems over neural methods is a matter of prioritizing correctness over coverage.

#### 2.2.4 Federative-Constraining (F-C): external guidance

F-C architectures function as *post-hoc* filters, using symbolic constraints to govern inference without impacting the training process. This pipeline uses a neural model in order to generate potential outputs which a symbolic module then validates or ranks using formal logic. A standard implementation of this is NeuroLogic Decoding (Lu et al., 2021); it prunes a language model's output space using predicate logic specifications that are entirely absent during the training stage. Such total architectural separation restricts interaction to output-level filtering decisions. The primary advantage here is implementational simplicity: layering a symbolic checker over a frozen, pretrained model yields formal output guarantees without requiring any weight updates.

However, F-C systems lack the iterative refinement that one finds in F-L architectures. The neural model generates and the symbolic module accepts/rejects. There is no feedback loop, and no opportunity for the symbolic constraints to guide the neural generation process toward better candidates. This limits the system's effectiveness when the constraint satisfaction score is low, because the system has to generate many candidates to find acceptable ones. As already mentioned, this category remains minimally explored compared to other approaches like F-R and F-L. The reason for this might be that the benefits of one-shot are weaker than iterative refinement or integrated reasoning.

### 2.3 Recent LLM-era developments: hybrid patterns

The distinction between injective and federative systems is becoming increasingly blurry, with recent integrations involving architectures that look federative by design but behave like tight feedback loops in practice. Logic-LM, LINC, and PAL illustrate this Neural → Symbolic gray area. In these systems, the LLM drafts a formal spec (SAT, SMT, or Python) and an external solver executes it. Strictly speaking, this is a federative system, because the solver remains a distinct, external entity. However, the coupling during inference is very tight. The cycle of generating a spec, executing it, and using the results to drive the next step mimics an F-L loop far more than it does standard, one-shot F-R reasoning.

DeepSeek-R1 (Guo et al., 2025) challenges the taxonomy in a different way. Extended reasoning emerges from pure reinforcement learning with no fine-tuning. DeepSeek-R1 used only Group Relative Policy Optimization (GRPO) during its training phase that involved no human-annotated reasoning examples. However, it managed to develop self-verification, reflection, and long chain-of-thought reasoning, achieving 79.8% on American Invitational Mathematics Examination 2024 (AIME), slightly improving upon OpenAI o1-1217. OpenAI's o1 and o3 models exhibit similar behavior: structured, multi-step reasoning that emerges from test-time compute scaling without an explicit symbolic module. These systems allocate variable computation at inference, producing internal chains of reasoning that resemble symbolic derivations but are implemented entirely within neural forward passes. The ability to transfer knowledge in these models is remarkable. Open-source distilled models show that reasoning patterns can effectively transfer to smaller systems. These systems resist categorization. They are primarily neural with no explicit symbolic component. However, they seem to involve emergent symbolic-like reasoning via test-time compute scaling. One way to conceptualize them is as implicit I-L systems in which symbolic structure emerges from optimization rather than being explicitly compiled. Alternatively, the taxonomy does not capture what is happening here, and these systems mark a boundary where the CL framework's applicability ends.

Large Concept Models (Barrault et al., 2024) push beyond current taxonomic boundaries in a different direction. They operate on sentence-level Sentence-Level multimOdal and Language-Agnostic Representations (SONAR) embeddings, not tokens. The 7B parameter model trained on roughly 2.7 trillion tokens demonstrates strong zero-shot cross-lingual generalization. Processing at the conceptual level seems to provide advantages for reasoning and long-document understanding.

However, LCM lacks formal symbolic integration, given that it operates in entirely learned embedding spaces. Thus, there is no explicit logic, no theorem provers, or formal rules. It adapts an architecture where multiple abstraction levels (tokens, sentences, and concepts) coexist. One could imagine symbolic constraints in these systems operating at the higher conceptual levels, while neural processing handles the lower-level perceptual mapping. This “hierarchical neuro-symbolic integration” represents a design space for which current taxonomies do not yet have a dedicated category. The preceding examples represent a group of systems at the boundaries of the CL taxonomy, and it is worth describing them in more detail. Tool-augmented LLMs, such as Toolformer (Schick et al., 2023), ReAct (Yao et al., 2022), and Reflexion (Shinn et al., 2023), superficially resemble federative architectures in that a neural LLM invokes external modules and incorporates their outputs. However, their external components, i.e., software APIs, search engines, or code interpreters, are not symbolic reasoners in the traditional NeSy sense, since they involve no formal logic, no theorem proving, and no probabilistic logic programming. We therefore consider these systems outside the CL taxonomy unless the external component performs formal reasoning. This is the case, for example, for PAL and SatLM, where the external component is a Python interpreter or a SAT solver operating over formally specified constraints. What makes them different is whether something external forces formal guarantees, and not whether something external is called. Similarly, test-time compute scaling models (DeepSeek-R1, OpenAI o1/o3) fall outside the taxonomy, as they contain no explicit symbolic component, whether internal or external. The CL framework was designed for systems that explicitly combine neural and symbolic components. Systems which simulate symbolic behavior through neural optimisation represent a distinct phenomenon, one that current architectural taxonomies, including ours, have not yet adequately captured.

### 2.4 Compositional generalization

So far, we have classified NeSy NLP systems by architecture and interaction mode. We now evaluate their performance across three dimensions: compositional generalization, reasoning and verification, and the patterns revealed by our taxonomy.

Compositional generalization refers to the ability of being able to produce more complex expressions from a set of basic vocabulary and, thus, being able to produce novel expressions that are the result of combining these basic vocabulary units, i.e., having learned “jump” and “twice,” correctly interpreting “jump twice” without having seen this specific combination during training. The SCAN benchmark tests this ability by requiring models to map instructions in natural language form to action sequences not seen during training. On these tasks, transformers fail. Lake and Baroni (2018) report that seq2seq models achieve only around 20% accuracy on length-based splits, with some configurations reaching near 0%. Modification of the training data can boost scores to near-perfect levels. However, this method sidesteps rather than solves the actual architectural problem (Patel et al., 2022). NeSy system provides a quite different result. The Compositional Program Generator achieves 100% accuracy on all SCAN test splits using just 14 training examples (Klinger et al., 2023). In comparison, the neural baseline requires nearly 17,000 examples.

The CPG learns composition rules rather than memorizing combinations. Given the rule for how “jump” combines with “twice,” it generalizes to “run twice,” “walk twice,” anything else combined with “twice.” Neural models have to see each combination. Lake and Baroni (2023) show similar results with Meta-Learning for Compositionality (MLC). The system reaches human-level systematic generalization on instruction-learning benchmarks, and manages to outperform frontier LLM models like GPT-4.

Compositional Generalization Challenge (COGS) exhibits the same pattern, with seq2seq models achieving 96%–99% in-distribution performance, but they reach only 16%–35% generalization accuracy (Kim and Linzen, 2020). They are able to memorize the training distribution effectively, but they are not able to generalize. The AM Parser, a NeSy compositional semantic parser with I-C architecture, reaches over 98% generalization accuracy. CPG hits 100% using only 22 examples (Klinger et al., 2023).

Neural approaches specifically adapted for the task can reach up to 88% on specific splits, but they require full training sets and lack systematic compositionality. They're fitting the test distribution, not learning compositional principles.

Compositional Freebase Questions (CFQ) presents a more nuanced picture. Standard Transformer models achieve 18% mean accuracy on Maximum Compound Divergence splits (Keysers et al., 2020) and pre-trained models like T5-11B reach 40.9%. Lastly, specialized architectures such as Hierarchical Poset Decoding achieve 67% (Guo et al., 2020). There's a strong negative correlation between compound divergence and accuracy. In general, models tend to perform better when train and test distributions share compositional patterns. They fail on novel combinations.

As a general conclusion, we could say that for tasks needing some sort of systematic compositional generalization, I-C architectures with explicit compositional structure seem to excel. The 1,000 × sample efficiency comes from learning composition rules rather than memorizing combinations. But this advantage seems limited to domains with explicit compositional structure involving commands, semantic parsing, query generation. Other aspects of natural language processing may not benefit equally. When the compositional structure isn't explicit or the task requires understanding beyond syntactic combination, the advantage disappears.

### 2.5 Reasoning and verification

Logical reasoning benchmarks reveal mixed success for Neural and NeSy systems, with performance gaps that appear less extreme than those seen in compositional generalization. The PRover system (Saha et al., 2020) achieves 87% accuracy on rule-based reasoning, yet this figure sinks to 65% when five-step reasoning chains are required. Such a decline illustrates how compounding errors consistently derail deeper inference. LogiQA 2.0 (Liu et al., 2023) presents a similarly difficult hurdle, testing categorical and conditional logic across 35,000 premise-hypothesis pairs. Top-performing models typically perform between 48 and 59% accuracy, trailing human benchmarks by a wide margin.

FraCaS is a dataset of 346 logical Natural Language Inferenced (NLI) cases that has been developed to check NLI systems' ability to reason logically. In this dataset, symbolic systems outperform neural ones. For example, type-theoretical systems achieve 81% (Bernardy and Chatzikyriakidis, 2021), while GPT-4 reaches 75% (Teng et al., 2023) and BERT-based models fall below 55% on monotonicity reasoning (Yanaka et al., 2019). However, while the 346-problem benchmark might enable targeted semantic evaluation, it is fairly limited given its very small size.

Vulnerabilities become more obvious under adversarial pressure; for instance, RoBERTa's accuracy on the Adversarial NLI (ANLI) dataset collapses from 73.8% in Round 1 to 44.4% by Round 3 (Nie et al., 2020). To mitigate this, VAULT (Kazoom et al., 2025) utilizes automated, validated adversarial generation to achieve non-trivial gains, pushing RoBERTa-Base to 80.95% on ANLI and boosting MultiNLI performance by 17.32%. Contrast test sets similarly expose model brittleness; systems maintaining 85%–95% accuracy on standard benchmarks often see 5%–25% performance drops following minimal perturbations (Gardner et al., 2020). As a case in point: ELECTRA-small slides from 89.9 to 72.5% on SNLI contrast sets, though contrast-augmented training can restore it to 85.5% (Sanwal, 2024). Such data indicates that models prioritize superficial pattern memorization over robust reasoning. Currently, neither injective nor federative architectures provide a clear advantage against adversarial or contrast challenges, as both paradigms are susceptible to spurious correlations. Architectural choice appears secondary to the training objective in these scenarios, implying that while the injective-federative split clarifies compositional generalization, it is less useful for addressing robustness in distribution shift.

### 2.6 Taxonomy-revealed patterns

Our classification system reveals a number of patterns across architectures. First, I-C systems dominate w.r.t. research volume, but they achieve small performance gains (1%–2%) on most tasks. The exception seems to be compositional generalization benchmarks where explicit structure provides decisive advantages (1,000 × sample efficiency on SCAN/COGS). F-R systems, on the other hand, present good performance increases (20%+ on deductive reasoning for DSR-LM, see Table 2), especially for tasks in which a separation of perception from logical inference is helpful. F-L systems, i.e., systems that combine LLMs with external verifiers, constitute a promising direction. None of these systems have been produced at foundational scale.

**Table 2:** Performance comparison across CL categories.

System	CL code	Task domain	Benchmark	Gain
Syntax-BERT	I-T	General NLU	GLUE	1%–2%
Syntax trees + SRL	I-C	Information extraction	SRL (gold parses)	2.6–3.5 F1
Tree-LSTMs	I-C	Sentiment	SST-5	+4.6%
LNN (NSQA)	I-C	KBQA	LC-QuAD	~3 F1
DeepProbLog	I-C	Visual QA	Compositional VQA	10 × sample eff.
CPG	I-C	Compositional gen.	SCAN	100% (14 examples)
DSR-LM	F-R	Deductive reasoning	Deduction tasks	+20%
Logic-LM	F-R	Logical reasoning	Logic benchmarks	+39.2%
LINC	F-R	Theorem proving	ProofWriter	+10% vs GPT-4
PAL	F-L	Arithmetic reasoning	GSM8K	+15%
SatLM	F-L	Constraint satisfaction	GSM (hard subset)	+23%
Rhyme system	F-L	Poetry generation	Greek rhyme	4% → 73.1%

Gains are over the respective non-symbolic or weaker baselines reported in each source.

I-T systems are ephemeral, given that the advantage of symbolic knowledge distilled during pre-training disappears with continued training (thus, no ongoing advantage). I-R systems are computationally expensive and superseded by models with cleaner F-R separation. F-G systems are symbolic data generators that are promising but underused compared to neural augmentation strategies.

Lastly, our taxonomy reveals a research gap. Federative systems are more successful but less represented. The field seems to be investing in marginal-gain injective approaches (I-C systems showing 1%–2% improvements), leaving stronger federative paradigms underexplored.

At this point, we need to say that the performance differences seen between injective and federative approaches might be due to differences in the domains of evaluation and not due to architectural differences. Injective systems are typically evaluated on broad NLU benchmarks (e.g., GLUE, SST) where marginal improvements of 1%–2% are the norm for any architectural modification. Looking at federative systems on the other hand, these are evaluated on tasks where the separation between perception and logical inference is much cleaner (e.g., ProofWriter, GSM8K, deductive reasoning). As such, symbolic components provide decisive advantages in these cases. Doing a fairer comparison would involve testing the same architectures on identical tasks or at least controlling for task characteristics. In this respect, what we observe points to a pattern, but it is not conclusive. Table 2 summarizes the findings across the CL categories.

This imbalance exists partly because of skepticism, but mostly because the field simply hasn't tested federative architectures at the scale of foundation models. Section 4 examines whether this gap stems from fundamental constraints or simply insufficient research investment, and it considers integrated systems that transcend current taxonomic boundaries.

## 3 Neural-symbolic NLP and linguistic theory

Linguistic theory remains a secondary concern for the systems detailed in Section 2, regardless of their technical merit. Optimization pressures frequently help neural networks bypass structural features, such as tree masks or dependency attributes, in favor of raw statistical shortcuts (Gururangan et al., 2018). This preference for dataset artifacts over principled compositional mechanisms highlights a persistent reliance on spurious correlations (Baroni, 2020). While probing studies investigate quantifier scope and monotonicity, they operate as *post-hoc* analyses that neither constrain model design nor contribute to theoretical linguistics. Recent analyses of deep learning's relationship with linguistic representation document this widening gap between neural advances and theoretical understanding (Lappin, 2021; Linzen and Baroni, 2021; Pavlick, 2022). Neither paradigm learns from the other.

This stalemate shows a number of gaps in contemporary NLP. All NeSy models we have today are bespoke, as they are designed for narrow tasks like visual reasoning or knowledge graph completion (Yi et al., 2018; Sarker et al., 2021). As such, they do not fulfill the foundational role that BERT or GPT play in purely neural NLP, i.e., a single pre-trained core adaptable system across diverse tasks with minimal fine-tuning. Moreover, linguistic theories are never pitted against each other under large-scale conditions. NLP imports isolated cues while calling results “linguistically informed”; theorists validate rules on carefully hand-crafted sentences. What is lacking is any mechanism for theories to reconfigure in response to large-scale evidence, or for neural systems to systematically incorporate theoretical insights (Linzen and Baroni, 2021; Pavlick, 2022). Finally, learning algorithms are conspicuously absent from mainstream formal linguistics. The disconnect between technical complexity and linguistic grounding in Section 2 remains a persistent issue. Even when architectures incorporate structural constraints, such as tree masks or dependency features, networks frequently abandon these properties during optimization in favor of raw statistical shortcuts (Gururangan et al., 2018). This tendency to exploit dataset artifacts and spurious correlations actively hinders the development of principled compositional mechanisms (Baroni, 2020). While probing studies have successfully identified model behaviors regarding quantifier scope and monotonicity, they operate primarily as *post-hoc* observations. Because they do not impose design-level constraints, these findings fail to either inform theoretical linguistics or reshape the underlying model architectures.

### 3.1 Existing attempts at linguistic theory integration

Work in which linguistic theories are combined with neural systems is sparse. However, a couple of proposals have been put forth. Perhaps, the most substantial one is Kogkalidis et al.'s (2020) work on Neural Proof Nets. There we find an implementation of differentiable proof nets for type-logical grammar using Sinkhorn networks. This system achieves end-to-end parsing from raw text to λ-calculus terms, with the proof-theoretic structure of linear logic preserved throughout. The architecture is genuinely NeSy (F-R), in that the neural components handle supertagging and axiom link prediction, and proof nets make sure that outputs are well-formed derivations with compositional semantic interpretations.

Other work integrates linguistic formalisms less deeply. For example, in Clark (2021); Tian et al. (2020) we find neural Combinatory Categorial Grammar (CCG) parsing, which is a combination of transformer-based supertaggers with symbolic CCG combinators. It produces SoA parsing accuracy results and preserves the transparent syntax-semantics interface of CCG. Zhou and Zhao present an HPSG neural parser (Zhou and Zhao, 2019), that introduces head-driven phrase structure into joint constituent-dependency parsing. This is an I-C system, in which the linguistic formalism informs architectural design rather than participating in inference. Monroe et al. (2017); Cohn-Gordon et al. (2018) propose systems that combine Bayesian pragmatic reasoning with neural models, using Rational Speech Acts (RSA) as differentiable layers, an I-L architecture with bidirectional flow between neural and probabilistic-symbolic components. Neural semantic parsing to λ-calculus (Dong and Lapata, 2016; Jia and Liang, 2016) produces Montague-style logical forms, but it treats them as output targets rather than integrating compositional semantics into the reasoning process.

Natural logic provides another way to integrate NeSy based on linguistic rules. An example of such a system can be found in Chen et al. (2021). The system presented there, NeuralLog makes use of a neural dependency parser to generate a dependency tree, then uses a deterministic Udep2Mono routine to label each node with monotonicity polarity, and then a symbolic module performs the reasoning. ProoFVer (Krishna et al., 2022) extends this approach to fact checking. The seq2seq neural model generates natural logic proofs as a sequence of lexical mutations, each labeled with a natural logic operator, and a deterministic finite automaton evaluates the resulting proof and provides a correctness assessment. This method achieves a 79.25% labeling accuracy for the FEVER method when the proofs are correct in their construction, i.e., the symbolic component ensures that the proof actually explains the solution.

### 3.2 The CL taxonomy as a guide for linguistically-informed architectures

Neural integration of major linguistic frameworks largely does not exist. The work in Section 3.1 covers most of what has been attempted. We believe the taxonomy proposed in this paper can function as a guide for future work in this direction, helping to match theoretical frameworks to an appropriate NeSy architecture A few examples illustrate the approach.

Type-theoretic semantics is based on type checking and proof construction, i.e., operations that can easily be fed to external proof assistants like Coq, Agda, Lean. An F-R architecture is an obvious choice here: neural components parse and extract semantic roles and the type checker verifies well-formedness. An F-L variant would involve a system where type-checking failures trigger neural reanalysis.

Incremental frameworks like Dynamic Syntax (Kempson et al., 2001) are a good fit for an F-L architecture, in which the neural part can process the next word, while the symbolic part performs the necessary steps for tree growth, and tree constraints in turn can shape neural predictions for what comes next.

Non-compositional frameworks like Construction Grammar (Goldberg, 1995) that use form-meaning pairs to organize knowledge are a better fit for an F-G architecture. Symbolic constructions are used to generate training data with guaranteed grammatical properties.

Recapitulating, we suggest identifying the framework's main contribution (verification, incrementality, and productivity), and then select the CL category that preserves it. This is a practical suggestion that can, in principle, help in embedding linguistic theories as symbolic modules of NeSy systems. This approach supports the choice of those parts of linguistic theories researchers regard as most significant for NeSy systems, or the specific tasks that NeSy systems are asked to solve.

## 4 Toward foundational neural-symbolic NLP

The taxonomic analysis in Section 2 shows that federative architectures consistently outperform injective ones, but the field keeps investing in injective designs to a greater extent than federative ones. These popular injective approaches deliver small gains, 1%–2% improvements, that often vanish with more training data. Furthermore, integration of linguistic theory across all methods is almost non-existent. Given this situation, we propose two research priorities.

First, federative architectures should be scaled up to foundation-model size. Right now, every successful federative system is small scale. FFNSL relies on modest neural components. DSR-LM and NLProlog tackle specialized reasoning tasks. Planning systems confined to structured action domains. We do not know whether keeping components separate still makes sense in a model with a billion parameters. The advantages visible at small scale, modular transparency, verified reasoning, specialized components, may or may not carry over. Interface overhead could balloon. Symbolic reasoners cut off from neural representations might struggle to learn. These are empirical questions that must be experimentally explored.

Second, suppose scaled federative approaches do work. The architectural divide that defines federation could still cap what neuro-symbolic integration achieves. Taxonomies available now, Kautz's interaction types, Lappin's architectural categories, the CL framework proposed here, all draw sharp lines between neural and symbolic sides. But some systems go beyond that line, for example, attention mechanisms with types built in, proof obligations threaded via gradient descent, unified substrates where representations mix distributional and logical properties from the start. Do these tightly coupled designs surpass federative ones in practice? We do not have the evidence to answer this question. Pure neural systems keep failing in familiar ways: hallucinating due to finite representational capacity (Peng et al., 2024), losing compositionality as complexity rises (Xu et al., 2024). Small fixes do not seem to help. Whether deeper integration addresses these failures, or creates different problems must be tested.

Work across the model categories from Section 2 should clarify which architectural models will be fruitful. The field needs federative systems built at scale, empirical checks on whether their advantages survive, and frank assessments of where architectural boundaries help and where they interfere. The taxonomic analysis here indicates what succeeds at small scale, and where research effort might not have produced the desired results. Building foundation-scale systems based on these findings is the required next move.

## 5 Conclusion

In this paper, we propose the Chatzikyriakidis-Lappin (CL) classification system (Table 1), that combines Lappin's architectural dimension along with five interaction modes of information flow (Teaching, Constraining, Generating, Reasoning, Loop) to systematically classify NeSy systems. The proposed framework shows an informative pattern already present in Lappin's taxonomy, namely that federative systems (F-R, F-L) consistently outperform injective approaches (I-C, I-T), but injective systems dominate published research. The CL taxonomy also reveals underexplored combinations. For example, the F-C category remains nearly empty, while I-L lacks large-scale implementations. The taxonomy has limitations. Systems with deeply integrated architectures where the neural-symbolic boundary dissolves entirely resist classification. It is true that deeply integrated systems exist as proposals. However, whether they offer any advantages over federative architectures remains an open question. Two research priorities emerge from our paper. First, the field should systematically scale federative architectures to foundation-model size in order to test whether advantages observed at small scale persist. Second, the architectural boundaries that define current taxonomies may themselves constrain what NeSy integration can achieve. The question of whether tighter integration helps or simply introduces new problems requires systematic investigation. Future work should determine which approaches prove viable at scale. The field needs implementations, empirical tests, and honest evaluation of where architectural choices matter and where they don't.

## Data Availability

The original contributions presented in the study are included in the article/supplementary material, further inquiries can be directed to the corresponding author.

## Footnotes

1 Generalization to out of domain test sets remains a serious problem for federative neural-symbolic symbolic models. Ranga (2025) shows that Cunnington et al. (2023)'s FFNSL does not succeed in learning symbolic rules for sentiment analysis from DNN output features in his experiments.
